# Efficacy of four internal fixation devices on femoral neck fractures in young adults: A systematic review and network meta-analysis

**DOI:** 10.1097/MD.0000000000040265

**Published:** 2024-11-08

**Authors:** Daotong Yuan, Zhimeng Zhang, Xu Wang, Wenjie Chang, Wenpeng Xie, Yongkui Zhang

**Affiliations:** a Shandong University of Traditional Chinese Medicine, Jinan City, Shandong Province, China; b Department of Orthopedic Surgery, Shandong University of Traditional Chinese Medicine Affiliated Hospital, Jinan City, Shandong Province, China.

**Keywords:** femoral neck fracture, femoral neck system, inverted triangle cannulated screws, medial buttress plate, percutaneous compression plate

## Abstract

**Background:**

The primary treatment of femoral neck fracture in young adults is internal fixation. The high complication rate after femoral neck fracture greatly affects the life of patients. There are many internal fixation devices for femoral neck fracture, but each has its advantages and disadvantages. Our aim was to determine the best internal fixation for young people with femoral neck fractures.

**Methods:**

We searched 5 databases from January, 2016 to December, 2023. Randomized controlled trials and cohort studies that met the inclusion criteria were assessed for quality using the RoB.2 and ROBINS-I scales, respectively. The network meta-analysis was conducted within a Bayesian framework utilizing a random effect model. Data analysis was performed using the “multinma” package within the R 4.2.0 software.

**Results:**

A network meta-analysis of 34 studies involving 2291 patients was conducted. Results indicated that the inverted triangular cannulated screws demonstrated the lowest intraoperative bleeding volume (surface under the cumulative ranking curve [SUCRA] = 0.8732) based on the SUCRA. The medial buttress plate (MBP) exhibited superior efficacy in improving the Harris hip score (SUCRA = 0.8465), reducing complications (SUCRA = 0.9251), and accelerating fracture healing time (SUCRA = 0.8111). Additionally, the femoral neck system was ranked highest in terms of operation time (SUCRA = 0.7749) and femoral neck shortening (SUCRA = 0.7933).

**Conclusion:**

This network meta-analysis findings indicated that MBP resulted in superior postoperative hip function, reduced complication rate, faster fracture healing time. Considering the good physical condition of young adults, surgeon may consider utilizing MBP to achieve improved postoperative outcomes.

## 1. Introduction

The femoral neck fracture occurs between the femoral head and the base of the femoral neck and is a common type of fracture, accounting for 3.6% of all fractures.^[1]^ High-energy injury in young people (<65 years old and >18 years old) is identified as the main cause of femoral neck fracture, and the incidence of complications is as high as 45%.^[2]^ High-energy injury often results in Pauwels type III femoral neck fractures, characterized by high shear force and instability.^[3]^ Treatment options for femoral neck fractures include internal fixation and joint replacement surgery, with internal fixation recommended for young adults to avoid the need for joint replacement revision.^[4–6]^ Various internal fixation devices are used in surgical procedures for femoral neck fractures, but some may not provide sufficient mechanical support during the healing process due to the unique anatomical structure and blood supply of the proximal femur.^[2,7]^ At present, prevalent internal fixation devices utilized in surgical procedures encompass various types such as multiple cannulated screws, femoral neck system (FNS), plate combined screws, dynamic hip screw (DHS), and DHS combined with a screw. Each of these internal fixation devices possesses distinct advantages and drawbacks. According to several meta-analyses, compared with inverted triangular cannulated screws (ITCS), other internal fixation devices only performed well in reoperation rate.^[8,9]^ While numerous biomechanical studies have analyzed the mechanical variances among these devices, the potential impacts of vascular injury and fracture fragments have often been overlooked.^[10,11]^

FNS has gained widespread adoption in surgical procedures as a new alternative to cannulated screws. Various meta-analyses have consistently demonstrated that FNS offered superior outcomes in terms of fracture healing time, femoral neck shortening, internal fixation failure, and Harris hip score compared to cannulated screws.^[12,13]^ Recent clinical and biomechanical studies have also shown the effectiveness of combining plates and cannulated screws for Pauwels type III fractures.^[10,14,15]^ While these fixation devices outperform ITCS, there is a lack of direct comparisons among these internal fixation devices, making it challenging for clinicians to select the most suitable surgical device.

In this study, we made a network meta-analysis of FNS, ITCS, and plate combined with cannulated screws in the treatment of femoral neck fracture to try to analyze the best internal fixation device and the difference of various internal fixation devices. In this study, we divided the treatment devices of plate combined with cannulated screw into medial buttress plate (MBP) and percutaneous compression plate (PCCP).

## 2. Methods

Our study was conducted n accordance with the Preferred Reporting Items for Systematic Reviews and Meta-analysis statement (Table S1, Supplemental Digital Content, http://links.lww.com/MD/N834).^[16]^ The review’s protocol was registered at PROSPERO (CRD42021293981).

### 2.1. Literature search

Two authors performed the literature search based on the Preferred Reporting Items for Systematic Reviews and Meta-analyses guidelines.^[16]^ Articles containing the efficacy of ITCS, FNS, MBP, or PCCP in the treatment of femoral neck fractures were searched in 5 online databases: PubMed, Embase, Cochrane Library, Web of Science, and China National Knowledge Infrastructure (CNKI). Among them, to ensure the quality of the included studies, we only included articles belonging to CSCD (the Chinese Science Citation Database) in CNKI database. To enhance the comprehensiveness of the search, specific search terms are detailed in Table S2, Supplemental Digital Content, http://links.lww.com/MD/N834. Articles were searched in databases from January 1, 2016 to December 1, 2023 without limitations to the publication language or status. The related literature and reviews were screened, and the references were manually reviewed to avoid omissions.

### 2.2. Inclusion and exclusion criteria

Two authors filtered all articles by reading the title and abstract independently. If the eligibility could not be determined from the abstract, the full article had to be evaluated. The inclusion and exclusion criteria were predetermined. The articles that met the following criteria were included: (1) adults under 65 years of age with unilateral femoral neck fracture (AO/OTA 31-B); (2) patients received ITCS, FNS, MBP, or PCCP treatment; (3) observation indexes include Harris hip score, complications, operation time, intraoperative blood loss, fracture healing time, femoral neck shortening, and so on; (4) randomized controlled trial or cohort study. The articles that met one of the following criteria were excluded: (1) patients with multiple fractures multiple traumas, pathological fracture, or reoperation; (2) participants included patients under 18 years old or over 65 years old; (3) studies using the same data set; (4) insufficient relevant data, reviews, duplicate articles, conference papers, and full texts not available.

### 2.3. Quality assessment and data extraction

For randomized controlled trials, we used RoB.2 for evaluation. For cohort studies, we used ROBINS-I as an evaluation tool.^[17,18]^ Two authors independently assessed these risks of bias, and any disputes regarding the assessment were independently assessed by a third author. Meanwhile, we used the CINaMe to rate the aggregate effect estimate of each major outcome.^[19]^ The main evidence quality of the results has 4 grades: high, medium, low, and very low. Baseline and outcome data for the included literature were independently recorded by both authors using the same data extraction tables, and contradictions were resolved through a discussion with the third author. The following information was extracted from the included articles: the name of the first author, publication year, research device, sample size and baseline data, intervention measures, treatment results, and so on.

### 2.4. Outcome measurements

In this study, the primary outcomes were Harris hip score and complications. The secondary outcomes were operation time, intraoperative bleeding volume, femoral head necrosis and nonunion, fracture healing time, and femoral neck shortening. Complications defined by us include: femoral head necrosis, nonunion, delayed union, internal fixation failure, and screw cutout.

### 2.5. Statistical analysis

Standard pair-wise and network meta-analysis based on Bayesian framework were used to analyzed ITCS, FNS, MBP, PCCP results.^[20,21]^ Continuous data were expressed using the weighted mean difference (MD), and the confidence interval (CI) was set to 95%. Dichotomous data were expressed using the odds ratio (OR) with 95% CI. The Hartung–Knapp–Sidik–Jonkman method was utilized for analyzing continuous data, while the Mantel–Haenszel method was used for dichotomous data. Heterogeneity was assessed using the Cochrane Q test (*P* < 10%) and Higgins *I*^2^ index (*I*^2^ > 50%). In cases where heterogeneity was observed in the analysis results, the random effects model was applied; otherwise, the fixed effects model was used. However, we only interpreted the results of the random-effects model as we believe that they can be generalized beyond the included studies.^[6,20]^ Data analysis was carried out by using “meta” and “gemtc” package under the software R 4.2.0. We ranked the therapeutic effect by the surface under the cumulative ranking curve (SUCRA), and the publication bias was evaluated through funnel plot and Egger test.^[22]^ *P* ≤ .05 was considered statistically significant.

## 3. Results

### 3.1. Literature characteristics

**Figure 1.**
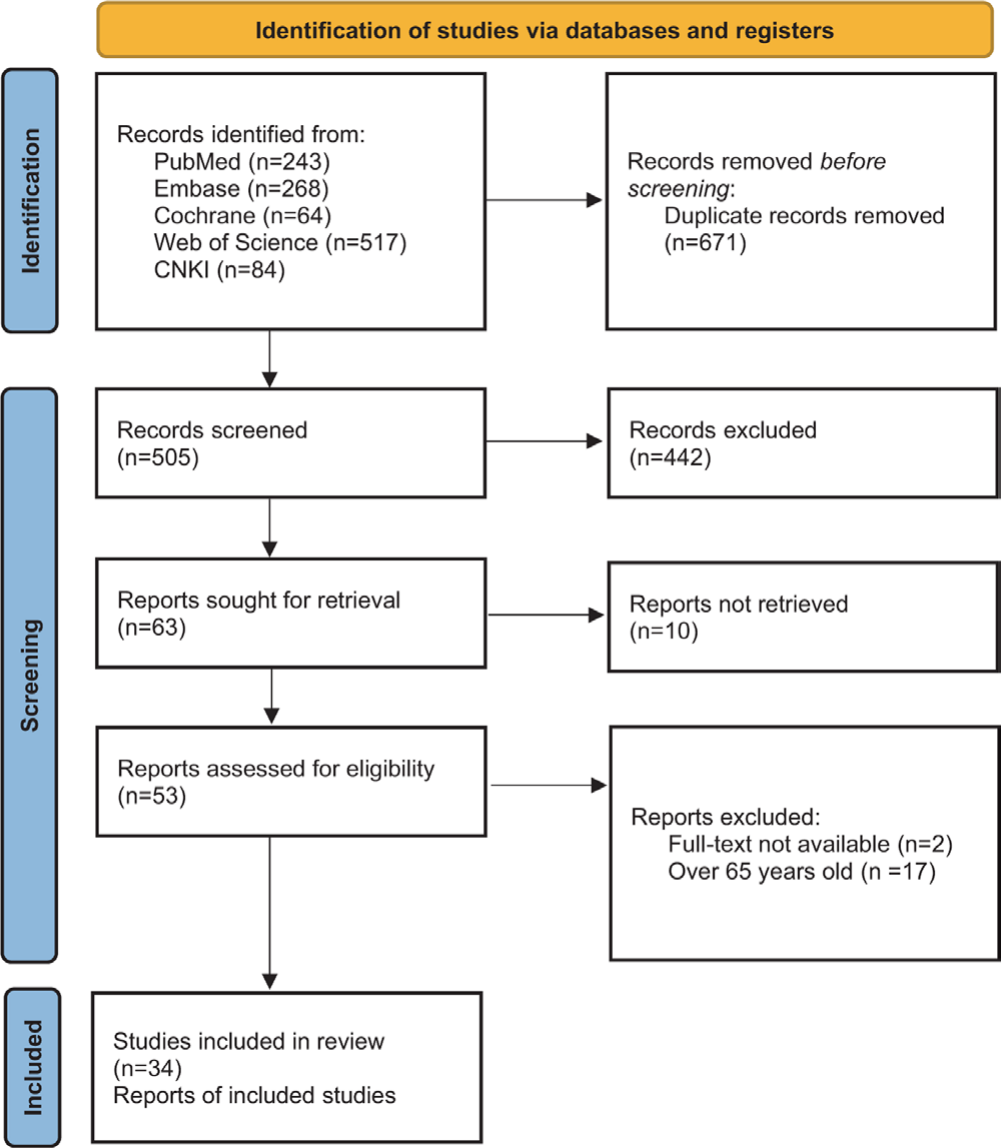
Flow chart of the article selection process.

A total of 1176 relevant studies were retrieved (PubMed, 243; Embase, 268; Cochrane Library, 64; Web of Science, 517; and CNKI, 84). After removing 671 duplicate articles, 442 studies were excluded based on their titles and abstracts. Following a thorough review of the full texts, 34 articles were selected for the final analysis (Fig. 1) encompassing a total of 2291 patients (FNS: 598, ITCS: 1220, MBP: 330, and PCCP: 143).^[15,23–55]^ Basic information regarding the included articles and their respective characteristics can be found in Table 1.

**Table 1:** Characteristics of included studies.

First author	Year	Intervention	Sample size	Mean age (year)	Female	Classification	Follow-up (mouth)	Outcome	Design
Wong Y	2022	FNS:ITCS	26/26	39.22/42.31	9/11	Garden/Pauwels	>12	2/3/4/5/6/7	RS
Yang J	2021	FNS:ITCS	28/31	22.5/22.6	11/14	Garden/Pauwels	3–14	1/2/3/4/5/6/7	RS
Yang Y	2021	FNS:ITCS	15/19	42.0/41.2	6/7	Garden/Pauwels	9–16	1/2/3/4/5/7	RS
Zhang F	2023	FNS:ITCS	25/27	45.20/49.30	11/16	Garden/Pauwels	6–32	1/2/3/4/5/6	RS
Zhou X	2021	FNS:ITCS	30/30	54.53/53.14	18/18	Pauwels	10–22	1/2/3/4/5/6	RS
Ge S	2022	FNS:ITCS	44/35	42.5/38.3	9/6	Pauwels	6~24	1/2/3/4/5/6/7	RS
Guy R	2023	FNS:ITCS	56/58	58.2/40.45	27/24	Garden/Pauwels	12–36	1/2/3/4/5/6/7	RS
He C	2021	FNS:ITCS	33/36	50.61/47.58	15/14	Garden	12–24	1/2/3/4/7	RS
Hu H	2021	FNS:ITCS	20/24	50.45/50.46	8/10	Garden/Pauwels	>6	1/2/3/4/5/6/7	RS
Lin H	2023	FNS:ITCS	27/31	57.9/57.6	20/22	Garden/Pauwels	6–48	1/2/3/4/5/6/7	RS
Su M	2023	FNS:ITCS	43/51	49.51/45.73	17/18	Garden/Pauwels	>12	1/2/3/4/5/6/7	RS
Tang Y	2021	FNS:ITCS	47/45	57.4/54.8	13/8	Garden/Pauwels	14–24	1/2/3/4/5/6/7	RS
Tao J	2023	FNS:ITCS	47/49	55.8/55.7	30/25	Garden	>12	1/2/4/5/6	RS
Wang L	2021	FNS:ITCS	14/10	42.23/42.04	4/4	Garden	6–20	1/3/4/5	RS
Yan S	2023	FNS:ITCS	22/27	51.00/46.15	10/13	Garden/Pauwels	>12	1/2/3/4/5/6	RS
Yang C	2021	FNS:ITCS	24/58	52/49	14/20	Garden/Pauwels	6–18	1/2/3/4/5/6	RS
Zhang B	2022	FNS:ITCS	31/34	51.8/50.4	17/18	Garden	>6	1/2/3/4/5/6	RS
Zhang Y	2022	FNS:ITCS	33/36	57.61/52.50	11/15	Garden	6	1/2/4/5/6	RS
Zheng H	2022	FNS:ITCS	18/18	47.0/46.4	8/8	Pauwels	>12	1/3/7	RS
Zhu Y	2022	FNS:ITCS	15/32	52.50/52.88	6/17	Garden/Pauwels	>6	1/2/3/4/5	RS
Gao C	2020	MBP:ITCS	25/30	43.03/41.32	13/15	Pauwels	12–15	1/2/3/4/5/6	RS
Huang Z	2022	MBP:ITCS	48/54	48.73/48.46	20/22	Garden	12–40	1/2/3/4/5/6/7	RS
Li Z	2022	MBP:ITCS	21/20	52.61/51.47	13/13	Pauwels	≥12	1/2/3/4/5/6	RS
Qin Y	2018	MBP:ITCS	30/30	35.50	19	Pauwels	12–18	1/2/3/4/5/6	RCT
Shen L	2023	MBP:ITCS	26/32	47.0/44.7	10/11	Pauwels	44.8/47.3*	1/2/3/4/5/6/7	RS
Sun X	2023	MBP:ITCS	32/36	36.62/35.86	13/15	Garden/Pauwels	12–42	1/2/3/4/5/6/7	RS
Wei F	2020	MBP:ITCS	35/41	43.7/45.1	15/19	Pauwels	16–18	1/2/6	RS
Zhao L	2023	MBP:ITCS	33/39	42.5/46.3	23/14	Pauwels	12–24	1/2/4/5/6	RS
Zou Y	2020	MBP:ITCS	58/55	43.62/43.56	25/21	Pauwels	9	1/2/3/4/5/6	RS
Ma C	2022	MBP:ITCS	22/47	43.86/40.28	5/12	Garden/Pauwels	>24	1/2/4/5/6/7	RS
Cui X	2023	PCCP:ITCS	31/37	45.2/40.8	13/19	Garden	>24	1/2/3/4/5/6/7	RS
Xu K	2020	PCCP:ITCS	45/55	45.8/43.3	21/25	Garden	24–56	1/2/3/6/7	RS
Ye J	2017	PCCP:ITCS	32/32	42.5/43.3	18/20	Garden	16–32	1/2/3/5/6	RS
Yin Q	2016	PCCP:ITCS	35/35	47.5/48.6	16/13	Garden	13–34	1/2/3/4/5/6	RCT

Outcomes: 1. Harris Hip score, 2. complication, 3. fracture healing time, 4. operation time, 5. intraoperative bleeding volume, 6. femoral head necrosis and non-union, 7. femoral neck shortening.

RCT = randomized controlled trial, RS = retrospective study.

* The mean of follow up time.

### 3.2. Quality assessment

Out of 34 studies reviewed, there were 2 randomized controlled trials and 32 cohort studies. The cohort studies were assessed for bias across 7 criteria: confounding, selection of participants into the study, classification of interventions, deviations from intended interventions, missing data, measurement of outcomes, selection of the reported result. For randomized controlled trial, we evaluated 6 aspects according to RoB.2. The assessment outcomes, detailing the bias risk and specific evaluations, are presented in Tables 2a and 2b.

**Table 2a:** The ROBINS-I scale of included studies.

Study		Pre-intervention		At intervention	Post-intervention				Overall risk of Bias
		Bias due to confounding	Bias in selection of participants into the study	Bias in classification of interventions	Bias due to deviations from intended interventions	Bias due to missing data	Bias in measurement of outcomes	Bias in selection of the reported result	
1	Wong Y (2022)	Low	Low	Low	Low	Low	Moderate	Low	Moderate
2	Yang J (2021)	Low	Low	Low	Low	Low	Moderate	Low	Moderate
3	Yang Y (2021)	Low	Low	Low	Low	Low	Moderate	Low	Moderate
4	Zhang F (2023)	Low	Low	Low	Low	Low	Moderate	Low	Moderate
5	Zhou X (2021)	Low	Low	Low	Low	Low	Moderate	Low	Moderate
6	Ge S (2022)	Low	Low	Low	Low	Low	Moderate	Low	Moderate
7	Guy R (2023)	Low	Low	Low	Low	Low	Moderate	Low	Moderate
8	He C (2021)	Low	Low	Low	Low	Low	Moderate	Low	Moderate
9	Hu H (2021)	Low	Low	Low	Low	Low	Moderate	Low	Moderate
10	Lin H (2023)	Low	Low	Low	Low	Low	Moderate	Low	Moderate
11	Su M (2023)	Low	Low	Low	Low	Low	Moderate	Low	Moderate
12	Tang Y (2021)	Low	Low	Low	Low	Low	Moderate	Low	Moderate
13	Tao J (2023)	Low	Low	Low	Low	Low	Moderate	Low	Moderate
14	Wang L (2021)	Low	Low	Low	Low	Low	Moderate	Low	Moderate
15	Yan S (2023)	Low	Low	Low	Low	Low	Moderate	Low	Moderate
16	Yang C (2021)	Low	Low	Low	Low	Low	Moderate	Low	Moderate
17	Zhang B (2022)	Low	Low	Low	Low	Low	Moderate	Low	Moderate
18	Zhang Y (2022)	Low	Low	Low	Low	Low	Moderate	Low	Moderate
19	Zheng H (2022)	Low	Low	Low	Low	Low	Moderate	Low	Moderate
20	Zhu Y (2022)	Low	Low	Low	Low	Low	Moderate	Low	Moderate
21	Gao C (2020)	Low	Low	Low	Low	Low	Moderate	Low	Moderate
22	Huang Z (2022)	Low	Low	Low	Low	Low	Moderate	Low	Moderate
23	Li Z (2022)	Low	Low	Low	Low	Low	Moderate	Low	Moderate
24	Shen L (2023)	Low	Low	Low	Low	Low	Moderate	Low	Moderate
25	Sun X (2023)	Low	Low	Low	Low	Low	Moderate	Low	Moderate
26	Wei F (2020)	Low	Low	Low	Low	Low	Moderate	Low	Moderate
27	Zhao L (2023)	Low	Low	Low	Low	Low	Moderate	Low	Moderate
28	Zou Y (2020)	Low	Low	Low	Low	Low	Moderate	Low	Moderate
29	Ma C (2022)	Low	Low	Low	Low	Low	Moderate	Low	Moderate
30	Cui X (2023)	Low	Low	Low	Low	Low	Moderate	Low	Moderate
31	Xu K (2020)	Low	Low	Low	Low	Low	Moderate	Low	Moderate
32	Ye J (2017)	Low	Low	Low	Low	Low	Moderate	Low	Moderate

**Table 2b:** Risk of bias assessment of the included randomized controlled trial studies according to the revised Cochrane risk of bias assessment tool.

	Random sequence generation (selection bias)	Allocation concealment (selection bias)	Blinding of participants and personnel (performance bias)	Blinding of outcome assessment (detection bias)	Incomplete outcome data (attrition bias)	Selective reporting (reporting bias)	Overall risk of bias
Qin Y (2018)	Low	Some concerns	Low	Low	Some concerns	Low	Some concerns
Yin Q (2016)	Low	Some concerns	Low	Low	Some concerns	Low	Some concerns

### 3.3. Primary outcomes

#### 3.3.1. Harris hip score

**Figure 2.**
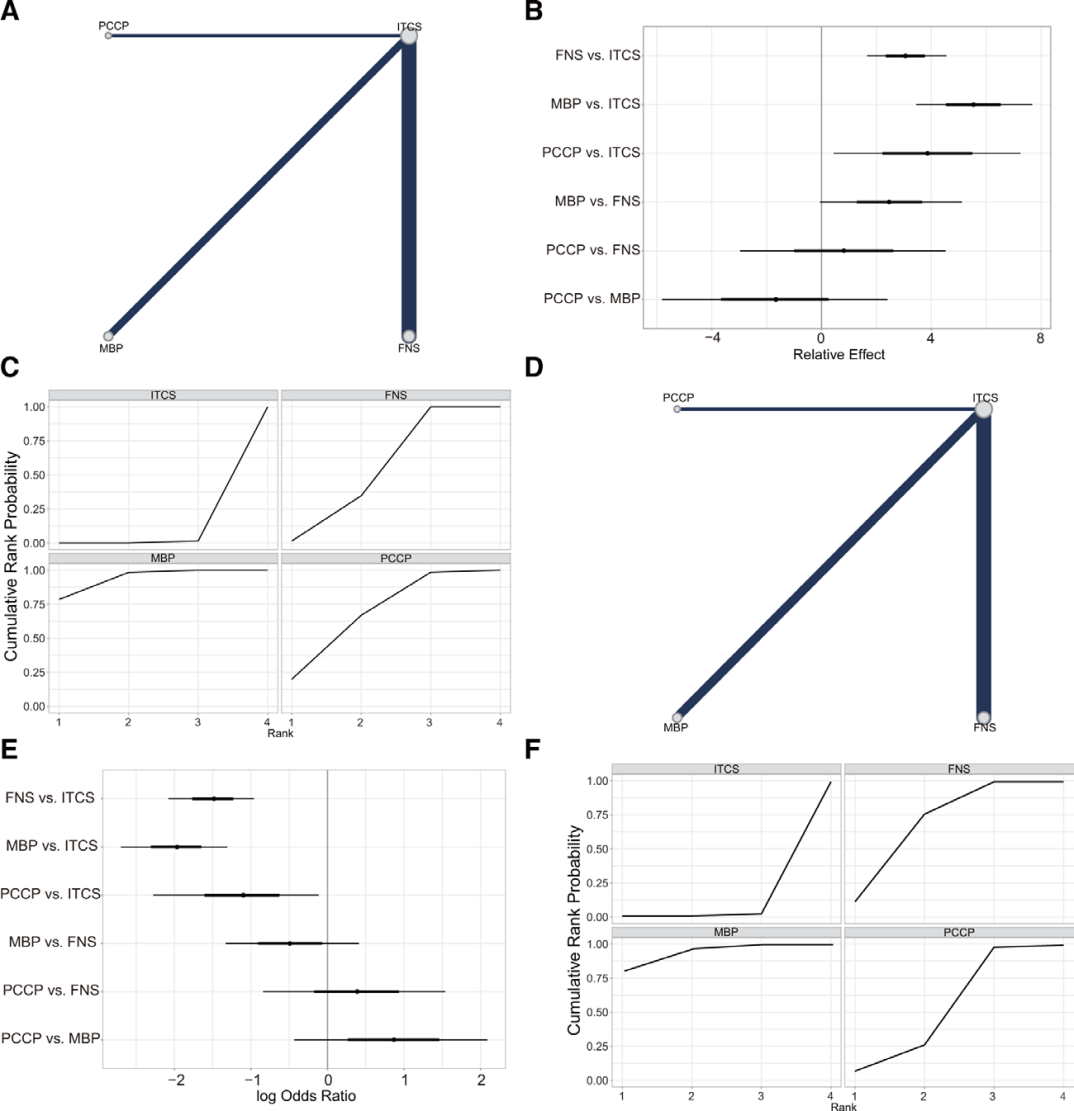
Results of the network meta-analysis of primary outcomes. (A) Network plot for Harris hip score. (B) Forest plot of final result for Harris hip score. (C) SUCRA plot for Harris hip score. (D) Network plot for complications. (E) Forest plot of network meta-analysis for complications. (F) SUCRA plot for complications. FNS = femoral neck system; ITCS = inverted triangle cannulated screws; MBP: medial buttress plate; PCCP = percutaneous compression plate; SUCRA = the surface under the cumulative ranking curve.

A total of 33 articles (n = 2239, 97.73%) included in the analysis reported the Harris hip score at the final follow-up assessment (Fig. 2A). In comparison to ITCS, FNS (MD = ‐3.05, 95% CI [‐4.56, −0.77]), MBP (MD = ‐4.89, 95% CI [‐7.17, ‐1.12]), and PCCP (MD = ‐4.28, 95% CI [‐7.61, −0.91]) were found to be significantly associated with an improvement in the Harris hip score. While the Harris hip score was slightly higher in MBP compared to FNS (MD = 1.84, 95% CI [‐0.77, 4.56]) and PCCP (MD = 0.61, 95% CI [‐3.37, 4.54]), this difference was not statistically significant (Fig. 2B, Table 3, and Fig. S1, Supplemental Digital Content, http://links.lww.com/MD/N833). The SUCRA values for ITCS, FNS, MBP, and PCCP were 0.002, 0.445, 0.847, and 0.706, respectively (Fig. 2C and Table 4). Based on the SUCRA values, MBP was identified as internal fixation devices that result in better hip function post-surgery, followed by PCCP, FNS, and ITCS.

**Table 3:** 95% confidence interval of pairwise comparison included in the study.

Treatment	Harris hip score	Complications	Operation time	Intraoperative bleeding volume	Femoral head necrosis and nonunion	Fracture healing time	Femoral neck shortening
ITCS vs FNS	‐3.05 (‐4.56, ‐0.77)*	1.98 (1.3, 2.70)*	1.05 (‐5.58, 7.93)	‐11.23 (‐22.70, ‐0.55)*	1.56 (0.86, 2.30)*	0.65 (0.38, 0.91)*	1.50 (0.84, 2.25)*
ITCS vs MBP	‐4.89 (‐7.17, ‐1.12)*	1.50 (0.96, 2.08)*	‐17.96 (‐26.07, ‐4.36)*	‐42.83 (‐57.20, ‐27.63)*	1.15 (0.53, 1.86)*	0.76 (0.32, 1.19)*	1.41 (0.48, 2.52)*
ITCS vs PCCP	‐4.28 (‐7.61, ‐0.91)*	1.12 (0.12, 2.28)*	‐3.39 (‐18.37, 10.70)	‐2.81 (‐20.00, 15.06)	0.69 (‐0.43, 1.91)	0.73 (0.14, 1.31)*	0.95 (‐0.16, 2.59)
FNS vs MBP	‐1.84 (‐4.56, 0.77)	0.48 (‐1.33, 0.41)	‐19.01 (‐30.28, ‐7.58)*	‐31.60 (‐49.5, ‐9.23)*	‐0.41 (‐1.34, 0.58)	0.11 (‐0.41, 0.62)	‐0.09 (‐1.23, 1.14)
FNS vs PCCP	‐1.24 (‐4.86, 2.37)	‐0.38 (‐1.54, 0.84)	‐4.44 (‐20.59, 11.25)	8.42 (‐11.30, 29.96)	‐0.88 (‐2.26, ‐0.70)*	0.07 (‐0.59, 0.71)	0.56 (‐1.88, 1.21)
MBP vs PCCP	0.61 (‐3.37, 4.54)	‐0.86 (‐2.09, 0.43)	14.57 (‐2.67, 30.80)	40.02 (17.70, 62.87)*	‐0.47 (‐1.83, ‐0.68)	‐0.03 (‐0.77, 0.72)	‐0.48 (‐1.92, 1.26)

FNS = femoral neck system, ITCS = inverted triangle cannulated screws, MBP = medial buttress plate, PCCP = percutaneous compression plate.

* *P* < .05.

**Table 4:** Treatment measures ranked by SUCRA method.

Treatment	Harris hip score	Complications	Operation time	Intraoperative bleeding volume	Femoral head necrosis and nonunion	Fracture healing time	Femoral neck shortening
ITCS	0.0023	0.0051	0.6854	0.8732	0.0373	0.0078	0.0168
FNS	0.4454	0.6272	0.7749	0.4092	0.6507	0.6340	0.7933
MBP	0.8465	0.9251	0.0164	0.0002	0.9012	0.8111	0.7253
PCCP	0.7058	0.4427	0.5232	0.7175	0.4108	0.5472	0.4645

FNS = femoral neck system, ITCS = inverted triangle cannulated screws, MBP = medial buttress plate, PCCP = percutaneous compression plate, SUCRA = the surface under the cumulative ranking curve.

#### 3.3.2. Complications

In all 32 studies analyzed, comprising a total of 2231 cases (97.38%), 415 cases of complications were reported, accounting for 18.6% of the total. Specifically, there were 314 cases (14.1%) in the ITCS group, 39 cases (1.7%) in the FNS group, 37 cases (1.7%) in the MBP group, and 25 cases (1.1%) in the PCCP group (Fig. 2D). Comparative analysis revealed that when compared to ITCS, FNS (OR = 1.98, 95% CI [1.3, 2.70]), MBP (OR = 1.50, 95% CI [0.96, 2.08]), and PCCP (OR = 1.12, 95% CI [0.12, 2.28]) were significantly associated with a reduction in complications. Among the FNS, MBP, and PCCP groups, the complication rate was marginally lower in the MBP group, although this difference did not reach statistical significance (Fig. 2E, Table 3, and Fig. S2, Supplemental Digital Content, http://links.lww.com/MD/N833). The SUCRA values for ITCS, FNS, MBP, and PCCP were 0.005, 0.627, 0.925, and 0.443, respectively (Fig. 2F and Table 4). Based on the SUCRA values for the different internal fixation devices, MBP exhibited the lowest complication rate, followed by FNS, PCCP, and ITCS.

### 3.4. Secondary outcomes

#### 3.4.1. Operation time

**Figure 3.**
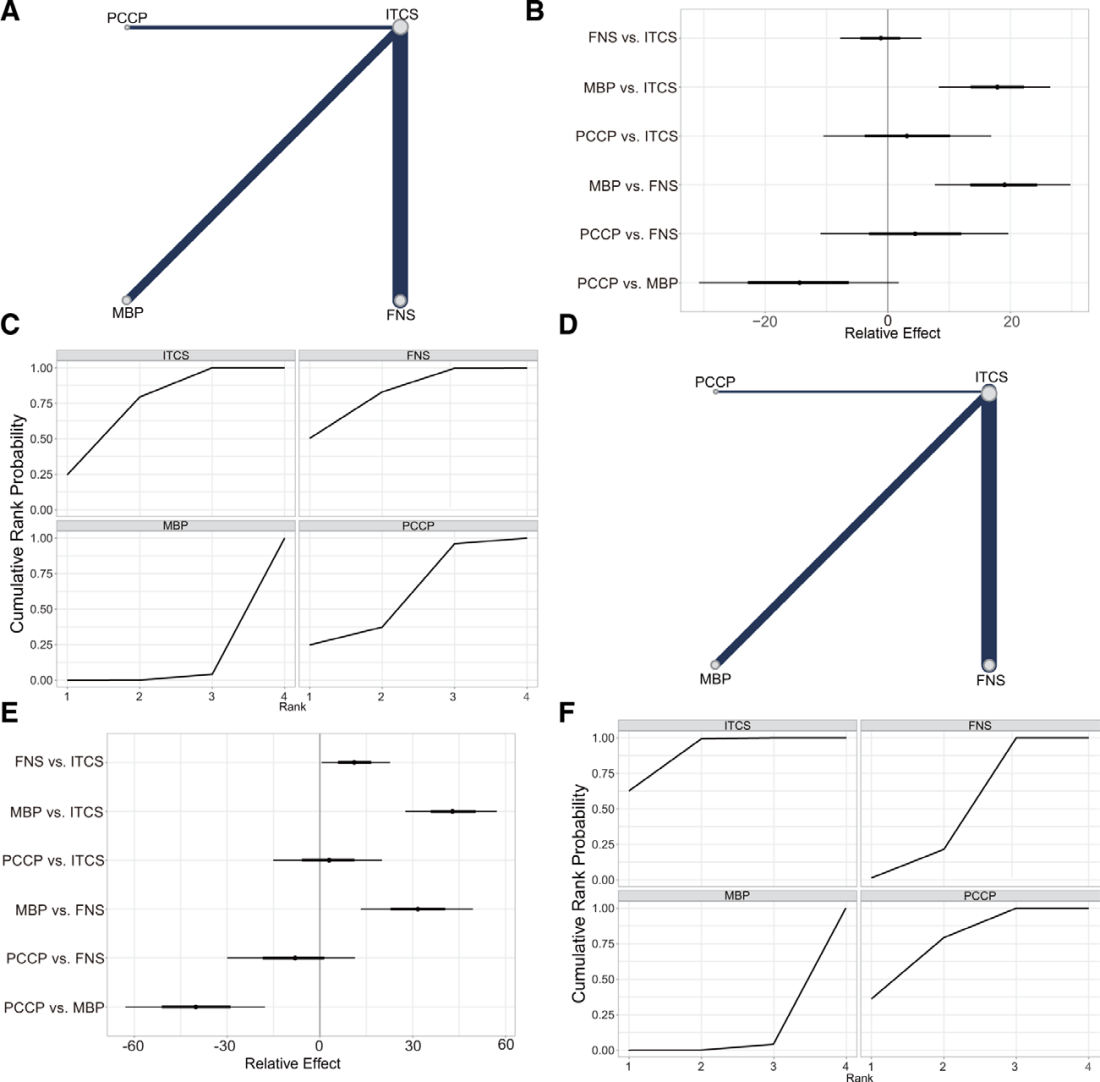
Results of the network meta-analysis of operation time and intraoperative bleeding volume. (A) Network plot for operation time. (B) Forest plot of final result for operation time. (C) SUCRA plot for operation time. (D) Network plot for intraoperative bleeding volume. (E) Forest plot of network meta-analysis for intraoperative bleeding volume. (F) SUCRA plot for intraoperative bleeding volume. ITCS: inverted triangle cannulated screws. FNS = femoral neck system; MBP = medial buttress plate; PCCP: = percutaneous compression plate; SUCRA = the surface under the cumulative ranking curve.

All 28 articles (n = 1891, 82.54%) reported the operation time (Fig. 3A). In comparison to ITCS, MBP showed a longer operation time (MD = ‐17.96, 95% CI [‐26.07, ‐4.36]), while there was no statistically significant difference between FNS (MD = 1.05, 95% CI [‐5.58, 7.93]) and PCCP (MD = ‐3.39, 95% CI [‐18.37, 10.70]) (Fig. 3B, Table 3, and Fig. S3, Supplemental Digital Content, http://links.lww.com/MD/N833). The SUCRA value of ITCS, FNS, MBP, and PCCP was 0.685, 0.775, 0.016, and 0.523, respectively (Fig. 3C and Table 4). Based on the SUCRA values of the different internal fixation devices, FNS had the shortest operation time, followed by ITCS, PCCP, and MBP.

#### 3.4.2. Intraoperative bleeding volume

All 29 articles (n = 1946, 84.94%) reported the intraoperative bleeding volume (Fig. 3D). Compared with ITCS, the intraoperative bleeding volume in MBP (MD = ‐42.83, 95% CI [‐57.20, ‐27.63]) and FNS (MD = ‐11.23, 95% CI [‐22.70, ‐0.55]) was more, while there was no significant difference in PCCP (MD = ‐2.81, 95% CI [‐20.00, 15.06]) (Fig. 3E, Table 3, and Fig. S4, Supplemental Digital Content, http://links.lww.com/MD/N833). The SUCRA value of ITCS, FNS, MBP, and PCCP was 0.873, 0.409, 0.0002, and 0.718, respectively (Fig. 3F and Table 4). According to the SUCRA values of various internal fixation devices, ITCS and PCCP had less intraoperative bleeding volume, followed by FNS and MBP.

#### 3.4.3. Femoral head necrosis and nonunion

**Figure 4.**
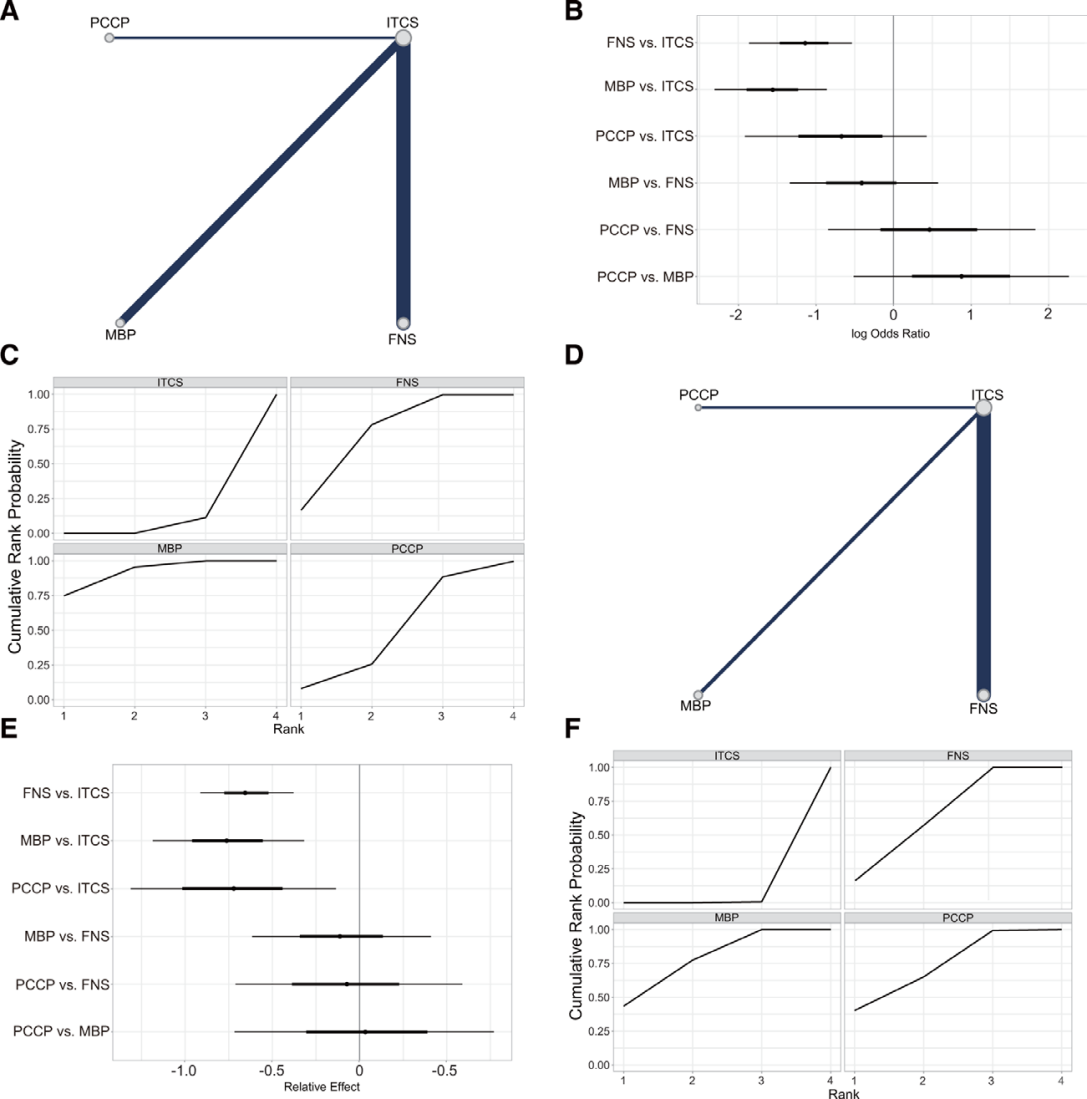
Results of the network meta-analysis of femoral head necrosis and nonunion and fracture healing time. (A) Network plot for femoral head necrosis and nonunion. (B) Forest plot of final result for femoral head necrosis and nonunion. (C) SUCRA plot for femoral head necrosis and nonunion. (D) Network plot for fracture healing time. (E) Forest plot of final result for fracture healing time. (F) SUCRA plot for fracture healing time. FNS = femoral neck system; ITCS = inverted triangle cannulated screws; MBP: medial buttress plate; PCCP: percutaneous compression plate; SUCRA: the surface under the cumulative ranking curve.

All 29 studies (n = 2086, 91.05%) reported 214 (10.3%) cases of femoral head necrosis and nonunion: 162 (7.8%) in the ITCS group, 24 (1.2%) in the FNS group, 19 (0.9%) in the MBP group, and 9 (0.4%) in the PCCP group (Fig. 4A). Compared with ITCS, FNS (OR = 1.56, 95% CI [0.86, 2.30]) and MBP (OR = 1.15, 95% CI [0.53, 1.86]) were significantly correlated with the decrease of femoral head necrosis and nonunion, while there was no significant difference in PCCP (OR = 0.69, 95% CI [‐0.43, 1.91]). Among FNS, MBP, and PCCP, the femoral head necrosis and nonunion rate was slightly lower in MBP group, but the difference was not statistically significant (Fig. 4B, Table 3, and Fig. S5, Supplemental Digital Content, http://links.lww.com/MD/N833). The SUCRA value of ITCS, FNS, MBP, and PCCP was 0.037, 0.651, 0.901, and 0.411, respectively (Fig. 4C and Table 4). According to the SUCRA values of internal fixation devices, MBP had the lowest he femoral head necrosis and nonunion rate, followed by FNS, PCCP, and ITCS.

#### 3.4.4. Fracture healing time

All 30 articles (n = 1981, 86.47%) reported the fracture healing time (Fig. 4D). Compared with ITCS, FNS (MD = 0.65, 95% CI [0.38, 0.91]), MBP (MD = 0.76, 95% CI [0.32, 1.19]), and PCCP (MD = 0.73, 95% CI [0.14, 1.31]) had a significantly shorter fracture healing time. However, there was no significant difference among FNS, MBP and PCCP (*P* > .05) (Fig. 4E, Table 3, and Fig. S6, Supplemental Digital Content, http://links.lww.com/MD/N833). The SUCRA value of ITCS, FNS, MBP, and PCCP was 0.008, 0.634, 0.811, and 0.547, respectively (Fig. 4F and Table 4). According to the SUCRA values of various internal fixation devices, MBP had the shortest fracture healing time, followed by FNS, PCCP, and ITCS.

#### 3.4.5. Femoral neck shortening

**Figure 5.**
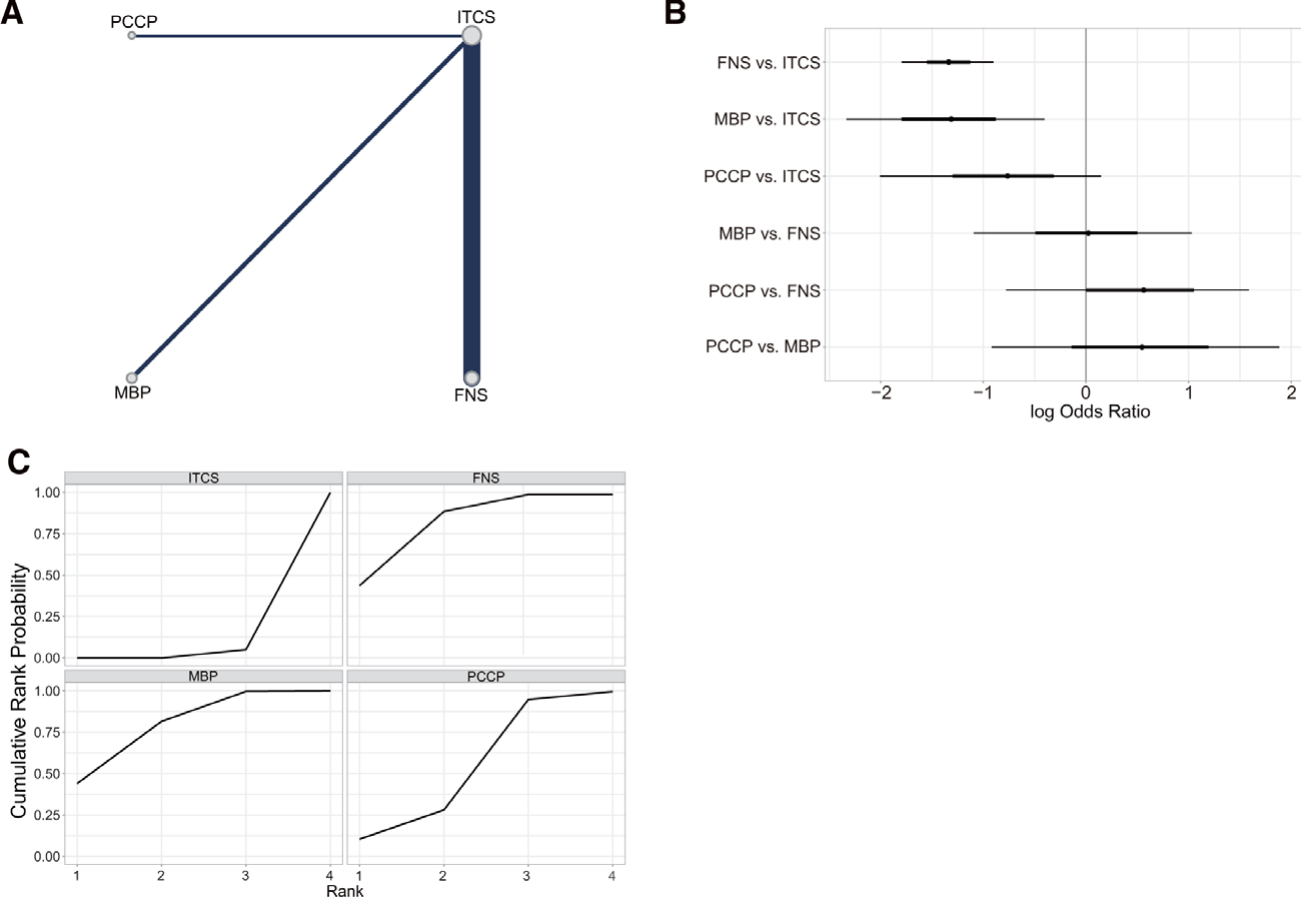
Results of the network meta-analysis of femoral neck shortening. (A) Network plot for fracture neck shortening. (B) Forest plot of final result for fracture neck shortening. (C) SUCRA plot for fracture neck shortening. FNS = femoral neck system; ITCS = inverted triangle cannulated screws; MBP = medial buttress plate; PCCP = percutaneous compression plate; SUCRA = the surface under the cumulative ranking curve.

All 20 studies (n = 1381, 60.28%) reported 455 (32.9%) cases of femoral neck shortening: 274 (19.8%) in the ITCS group, 157 (11.4%) in the FNS group, 13 (0.9%) in the MBP group, and 11 (0.8%) in the PCCP group (Fig. 5A). Compared with ITCS, FNS (OR = 1.51, 95% CI [0.84, 2.25]) and MBP (OR = 0.65, 95% CI [0.38, 0.91]) were significantly correlated with the decrease of femoral neck shortening rate, while there was no significant difference in PCCP. Among FNS, MBP and PCCP, the femoral neck shortening rate was slightly lower in FNS group, but the difference was not statistically significant (Fig. 5B, Table 3, and Fig. S7, Supplemental Digital Content, http://links.lww.com/MD/N833). The SUCRA value of ITCS, FNS, MBP, and PCCP was 0.017, 0.793, 0.725, and 0.465, respectively (Fig. 5C and Table 4). According to the SUCRA values of various internal fixation devices, FNS and MBP had lower femoral neck shortening rate, followed by PCCP and ITCS.

### 3.5. Inconsistency and publication bias

**Figure 6.**
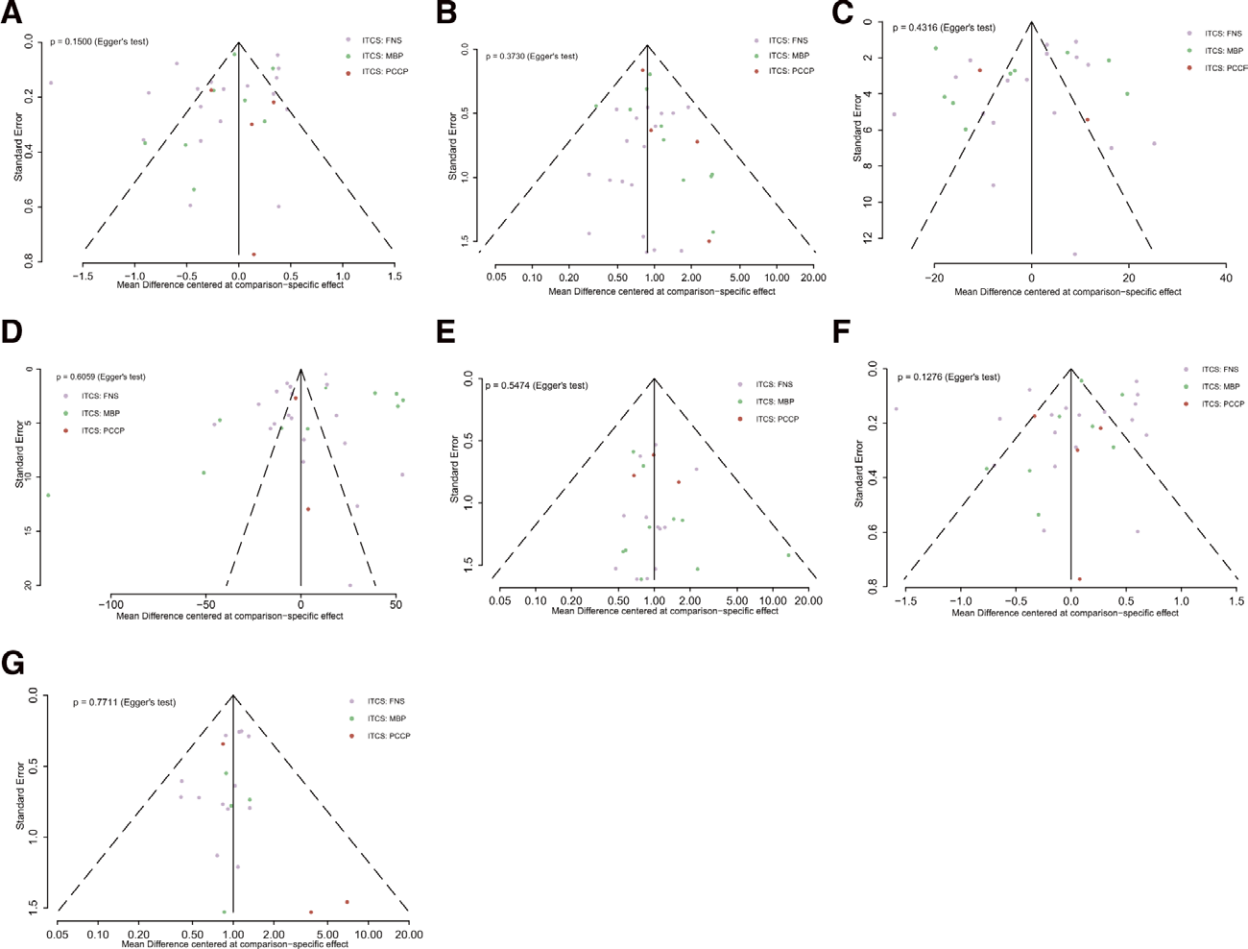
Funnel plot for outcomes. (A) Harris hip score, (B) complications, (C) operation time, (D) intraoperative bleeding volume, (E) femoral head necrosis and nonunion, (F) fracture healing time, (G) femoral neck shortening.

The global inconsistency was identified through an unrelated mean effect model, with findings indicating no overall inconsistency across all indicators (Table 5). Additionally, an assessment of publication bias for each outcome was conducted, revealing no apparent asymmetry in the funnel plot. Further evaluation of bias risk using the Egger test demonstrated that the bias risk was not statistically significant (Fig. 6A–G).

**Table 5:** Inconsistency test of network meta-analysis.

	Consistency model	Unrelated mean effect model
Harris Hip score	123.0	122.8
Complication	110.1	110.8
Operation time	109.0	109.5
Intraoperative bleeding volume	121.0	120.1
Femoral head necrosis and nonunion	98.0	99.0
Fracture healing time	112.7	112.9
Femoral neck shortening	61.8	62.1

### 3.6. Level of evidence

The levels of evidence were evaluated utilizing CINeMa, with the majority of confidence ratings falling within the low to moderate range, and a few being categorized as very low (Table 6).

**Table 6:** Evidence level results evaluated using CINaMe.

Comparison	Harris hip score	Complications	Operation time	Intraoperative bleeding volume	Femoral head necrosis and nonunion	Fracture healing time	Femoral neck shortening
ITCS: FNS	Moderate	Moderate	Moderate	Moderate	Moderate	Moderate	Moderate
ITCS: MBP	Moderate	Moderate	Moderate	Moderate	Moderate	Moderate	Moderate
ITCS: PCCP	Moderate	Moderate	Moderate	Moderate	Moderate	Moderate	Moderate
FNS: MBP	Low	Moderate	Low	Low	Low	Low	Very low
FNS: PCCP	Low	Low	Very Low	Low	Very low	Low	Low
MBP: PCCP	Very low	Very low	Low	Low	Very low	Low	Low

## 4. Discussion

The use of ITCS was widely recognized as the preferred treatment for femoral neck fractures in young adults.^[56]^ However, there are several internal fixation devices that exhibit superior qualities to ITCS in certain aspects.^[57]^ This study represents the initial network meta-analysis conducted on a range of internal fixation devices for femoral neck fractures in young adults. Previous research primarily focused on comparing cannulated screws with other internal fixation devices, yielding varying and contentious results.^[8,9,12,46,58–65]^ While it was established which internal fixation devices outperformed cannulated screws, the comparative advantages and disadvantages of alternative devices remained unclear. Factors such as injury mechanism, patient’s physical condition and surgical approach will significantly affect postoperative recovery.^[57,66]^ Among the commonly used internal fixation devices, DHS was excluded from this study because the previous research results showed that FNS was superior to DHS.^[67]^ The purpose of this study was to compare the efficacy of internal fixation devices other than ITCS by using a network meta-analysis, aiming to supplement the lack of direct comparison of various internal fixations in clinical research. We hope this study could help us to choose surgical device according to the advantages and disadvantages of different internal fixation devices.

Among the 4 types of internal fixation devices, the MBP provided patients with improved postoperative hip joint function (as assessed by the Harris hip score), shorter fracture healing time, reduced degree of femoral neck shortening, and lower incidence of complications. However, the MBP demonstrated poor performance in terms of operation time and intraoperative bleeding volume. The ITCS exhibited the least intraoperative blood loss, while the FNS had the shortest operation time. In this analysis, the PCCP showed suboptimal performance, despite being frequently used in previous studies for unstable femoral neck fractures.^[68]^

Harris hip score is used to evaluate the postoperative hip joint function, which is an important result of evaluating the internal fixation effect of femoral neck fracture. It is evaluated from 4 aspects: pain, function, deformity, and range of motion of hip joint. Harris hip score was related to many factors, such as fracture healing time, femoral head necrosis, shortening of femoral neck, change of neck shaft angle, and so on.^[69–71]^ Although functional training helped patients recover hip function as early as possible, the difference of Harris hip score after long-term operation was mainly related to complications.^[70]^ In this study, with the superior performance in terms of fracture healing time, complication rate and femoral neck shortening, MBP demonstrated the highest Harris hip score.

The stability of fracture fixation is closely associated with the implant materials and the quality of reduction. The physiological structure of the femoral neck enables it to bear weight, resist significant shear and rotational forces. An ideal internal fixation device should possess adequate stability, shear resistance, and rotational resistance. MBP could withstand pressure and shear forces at the fracture ends. Cannulated screw and medial buttress plate were combined in a clamp-like manner to convert stress at the fracture end into pressure between the fracture ends, maintaining stability of fracture fragments and providing strong fixation strength.^[72]^ MBP offered an additional way to distribute stress evenly, withstand higher shear forces, and maintain a favorable mechanical environment when the affected limb was active.^[73]^ Li et al observed a significant reduction in displacement of the femoral neck and internal fixation with the addition of medial buttress plates, stabilizing the position of fracture fragments.^[74]^ Compared to single hollow screws and DHS, medial buttress plates further reinforce the connection at the medial end of femoral neck fractures, enhancing rotational resistance.^[73,75]^

The blood supply to the femoral head and neck is unique, which is a major reason for the high rate of avascular necrosis of the femoral head after femoral neck fractures. Many orthopedic surgeons opt for closed reduction due to concerns about compromising the blood supply during open reduction. However, Mei et al have demonstrated that this notion was incorrect.^[76]^ The medial femoral circumflex artery provides nearly 80% of the blood supply to the femoral neck and head. The superior retinacular artery was often injured in fractures because it was close to the femoral neck, while the inferior retinacular artery was not easily damaged and mainly provides blood supply to the weight-bearing part of the femoral head. Studies have shown that the inferior supporting artery was located at the posterior position of the femoral neck from 7:00 to 8:00, so placing the medial plate in the 6:00 direction would not damage the important blood vessels.^[76,77]^ Collinge et al found that positioning medial buttress plate at the medial midline of the femoral head carried a lower risk of avascular necrosis compared to anterior or posterior placements.^[72]^ In comparison to closed reduction, open reduction offered superior quality of realignment, reduced increase in intra-articular pressure of the hip joint, and less repeated damage to surrounding blood vessels caused by closed reduction.^[78,79]^ Compared with these benefits, the disadvantages of MBP, such as longer operation time and more intraoperative blood loss, were acceptable.^[76]^

This study has some limitations. Firstly, most of the included literatures are retrospective cohort studies. Although the bias risk of cohort studies is moderate, it may still affect the quality of evidence. Secondly, the network meta-analysis did not constitute a loop structure, so that some comparisons lacked direct or indirect comparisons and the level of evidence was low. Thirdly, there is variation in the distribution of fracture classifications among the literature reviewed, and the duration of follow-up ranges from 6 months to 4 years, potentially influencing the outcomes. Given the aforementioned issues, it is necessary to undertake multicenter randomized controlled trials to analysis the comparative effectiveness of various internal fixation devices across different types of fractures.

## 5. Conclusion

The results of meta-analysis showed that MBP had the best performance in terms of hip Harris score, complications, and fracture healing time. FNS had the shortest operation time, the least femoral neck shortening, and ITCS had the least intraoperative blood loss. After a comprehensive evaluation of the patient’s physical condition, we recommend MBP as the preferred internal fixation device for femoral neck fractures in young adults.

## Author contributions

**Conceptualization:** Daotong Yuan, Yongkui Zhang.

**Data curation:** Daotong Yuan, Zhimeng Zhang, Xu Wang, Wenjie Chang.

**Formal analysis:** Daotong Yuan, Zhimeng Zhang, Xu Wang.

**Investigation:** Daotong Yuan, Zhimeng Zhang, Xu Wang, Wenjie Chang.

**Project administration:** Wenpeng Xie, Yongkui Zhang.

**Supervision:** Wenpeng Xie, Yongkui Zhang.

**Visualization:** Zhimeng Zhang, Wenjie Chang.

**Writing – review & editing:** Daotong Yuan, Wenjie Chang, Yongkui Zhang.

## Supplementary Material


