# Supplementary material for: Efficacy of four internal fixation devices on femoral neck fractures in young adults: A systematic review and network meta-analysis
Source: Medicine (Baltimore). 2024 Nov 8;103(45):e40265. doi: 10.1097/MD.0000000000040265 (PMC11557037; doi:10.1097/MD.0000000000040265)
Supplement: Supplementary file 2 [file medi-103-e40265-s002.pdf]

Study	ITCS Total	ITCS Mean	ITCS SD	FNS Total	FNS Mean	FNS SD	MD	95%−CI	Weight (Fixed)	Weight (Random)
Yang J−2021	31	88.40	6.20	28	92.40	5.50	−4.00	[−6.99; −1.01]	1.8%	4.6%
Yang Y−2021	19	90.80	3.30	15	89.80	2.50	1.00	[−0.95; 2.95]	4.1%	5.7%
Zhang F−2023	27	87.07	4.71	25	91.04	2.64	−3.97	[−6.03; −1.91]	3.7%	5.6%
Zhou X−2021	30	82.37	7.52	30	86.16	7.26	−3.79	[−7.53; −0.05]	1.1%	3.9%
Ge S−2022	35	89.10	2.70	44	90.40	1.60	−1.30	[−2.31; −0.29]	15.3%	6.6%
Guy R−2023	58	84.28	2.24	56	87.80	1.92	−3.52	[−4.28; −2.76]	26.8%	6.7%
He C−2021	36	88.44	5.91	33	90.42	4.82	−1.98	[−4.52; 0.56]	2.4%	5.1%
Hu H−2021	24	81.92	8.34	20	85.90	5.98	−3.98	[−8.22; 0.26]	0.9%	3.4%
Lin H−2023	31	82.80	8.10	27	87.60	5.50	−4.80	[−8.33; −1.27]	1.3%	4.1%
Su M−2023	51	84.59	3.62	43	86.56	2.79	−1.97	[−3.27; −0.67]	9.3%	6.3%
Tang Y−2021	45	84.40	3.20	47	88.90	4.30	−4.50	[−6.04; −2.96]	6.6%	6.1%
Tao J−2023	49	79.90	8.30	47	81.30	9.70	−1.40	[−5.02; 2.22]	1.2%	4.0%
Wang L−2021	10	89.20	2.50	14	91.00	3.30	−1.80	[−4.12; 0.52]	2.9%	5.3%
Yan S−2023	27	88.18	6.19	22	90.07	5.54	−1.89	[−5.18; 1.40]	1.5%	4.3%
Yang C−2021	58	85.00	9.00	24	94.70	1.60	−9.70	[−12.10; −7.30]	2.7%	5.2%
Zhang B−2022	34	93.10	5.90	31	97.00	4.10	−3.90	[−6.35; −1.45]	2.6%	5.2%
Zhang Y−2022	36	78.67	3.72	33	84.61	3.42	−5.94	[−7.62; −4.26]	5.5%	6.0%
Zheng H−2022	18	92.70	3.30	18	95.00	2.20	−2.30	[−4.13; −0.47]	4.7%	5.8%
Zhu Y−2022	32	87.03	3.00	15	86.80	2.61	0.23	[−1.45; 1.91]	5.6%	6.0%
**Fixed effect model**							**−2.87**	**[−3.27; −2.47]**	**100.0%**	**.**
**Random effects model**	**651**			**572**			**−3.07**	**[−4.23; −1.91]**	**.**	**100.0%**

Heterogeneity: $I^2 = 61\%$, $\tau^2 = 4.5542$, $p < 0.01$

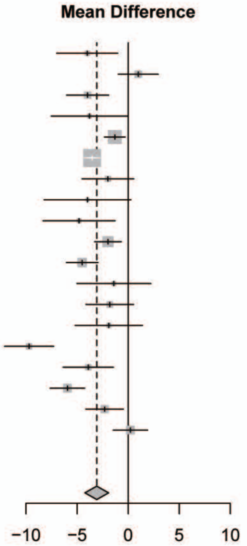

Study	ITCS Total	ITCS Mean	ITCS SD	MBP Total	MBP Mean	MBP SD	MD	95%−CI	Weight (Fixed)	Weight (Random)
Gao C−2020	30	88.17	3.36	25	92.04	3.72	−3.87	[−5.76; −1.98]	3.2%	11.1%
Huang Z−2022	54	82.47	6.33	48	85.60	4.36	−3.13	[−5.22; −1.04]	2.7%	11.0%
Li Z−2022	20	61.84	7.73	21	73.92	8.25	−12.08	[−16.97; −7.19]	0.5%	8.6%
Qin Y−2018	30	82.47	6.63	30	88.37	3.21	−5.90	[−8.53; −3.27]	1.7%	10.6%
Shen L−2023	32	81.70	16.40	26	85.00	13.80	−3.30	[−11.07; 4.47]	0.2%	6.1%
Sun X−2023	36	7.93	0.80	32	8.14	0.77	−0.21	[−0.58; 0.16]	83.2%	11.7%
Wei F−2020	41	83.40	9.60	35	91.50	8.40	−8.10	[−12.15; −4.05]	0.7%	9.4%
Zhao L−2023	39	83.30	9.40	33	92.60	5.80	−9.30	[−12.85; −5.75]	0.9%	9.8%
Zou Y−2020	55	61.98	7.14	58	73.96	8.11	−11.98	[−14.79; −9.17]	1.5%	10.4%
Ma C−2022	47	92.51	2.80	22	92.91	2.93	−0.40	[−1.86; 1.06]	5.4%	11.3%
**Fixed effect model**							**−0.89**	**[−1.23; −0.55]**	**100.0%**	**.**
**Random effects model**	**384**			**330**			**−5.63**	**[−8.79; −2.47]**	**.**	**100.0%**

Heterogeneity: $I^2 = 64\%$, $\tau^2 = 17.3097$, $p < 0.01$

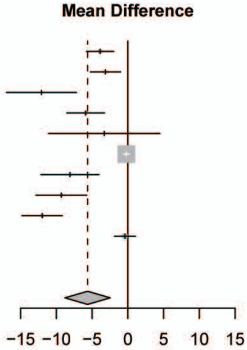

Study	ITCS Total	ITCS Mean	ITCS SD	FNS Total	FNS Mean	FNS SD	MD	95%−CI	Weight (Fixed)	Weight (Random)
Cui X−2023	37	86.06	7.76	31	91.45	5.60	−5.39	[−8.57; −2.21]	22.0%	24.5%
Xu K−2020	55	91.41	6.10	45	90.68	8.05	0.73	[−2.12; 3.58]	27.4%	25.4%
Ye J−2017	32	89.51	6.37	32	92.29	6.14	−2.78	[−5.85; 0.29]	23.7%	24.8%
Yin Q−2016	35	83.70	6.20	35	92.20	6.10	−8.50	[−11.38; −5.62]	26.9%	25.3%
**Fixed effect model**							**−3.93**	**[−5.42; −2.43]**	**100.0%**	**.**
**Random effects model**	**159**			**143**			**−3.98**	**[−10.24; 2.29]**	**.**	**100.0%**

Heterogeneity: $I^2 = 86\%$, $\tau^2 = 13.3482$, $p < 0.01$

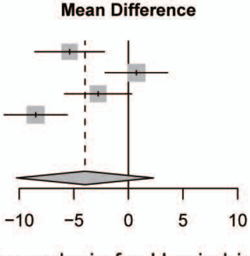

Figure S1 Forest plot of standard pair-wise meta-analysis for Harris hip score.

Study	ITCS Events	ITCS Total	FNS Events	FNS Total	OR	95%−CI	Weight (Fixed)	Weight (Random)
Wong Y−2022	7	26	3	26	2.82	[0.64; 12.44]	6.9%	6.9%
Yang J−2021	4	31	0	28	9.33	[0.48; 181.51]	1.7%	1.7%
Yang Y−2021	1	19	0	15	2.51	[0.10; 66.20]	1.4%	1.4%
Zhang F−2023	9	27	4	25	2.62	[0.69; 9.98]	8.6%	8.6%
Zhou X−2021	8	30	2	30	5.09	[0.98; 26.43]	5.6%	5.6%
Ge S−2022	7	35	7	44	1.32	[0.42; 4.20]	11.4%	11.4%
Guy R−2023	14	58	5	56	3.25	[1.08; 9.73]	12.7%	12.7%
He C−2021	6	36	1	33	6.40	[0.73; 56.32]	3.2%	3.2%
Hu H−2021	12	24	3	20	5.67	[1.31; 24.53]	7.1%	7.1%
Lin H−2023	5	31	1	27	5.00	[0.55; 45.80]	3.1%	3.1%
Su M−2023	23	51	4	43	8.01	[2.49; 25.74]	11.2%	11.2%
Tang Y−2021	8	45	5	47	1.82	[0.55; 6.04]	10.6%	10.6%
Tao J−2023	6	49	2	47	3.14	[0.60; 16.41]	5.6%	5.6%
Yan S−2023	10	27	1	22	12.35	[1.43; 106.34]	3.3%	3.3%
Yang C−2021	3	58	0	24	3.09	[0.15; 62.13]	1.7%	1.7%
Zhang B−2022	4	34	1	31	4.00	[0.42; 37.91]	3.0%	3.0%
Zhang Y−2022	1	36	0	33	2.83	[0.11; 71.94]	1.5%	1.5%
Zhu Y−2022	1	32	0	15	1.48	[0.06; 38.37]	1.4%	1.4%
**Fixed effect model**		**649**		**566**	**3.44**	**[2.33; 5.09]**	**100.0%**	**.**
**Random effects model**					**3.44**	**[2.53; 4.69]**	**.**	**100.0%**

Heterogeneity: $I^2 = 0\%$, $\tau^2 = 0$, $p = 0.93$

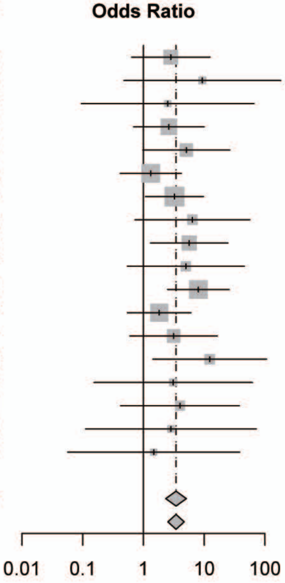

Study	ITCS Events	ITCS Total	MBP Events	MBP Total	OR	95%−CI	Weight (Fixed)	Weight (Random)
Gao C−2020	11	30	1	25	13.89	[1.65; 117.36]	3.9%	7.8%
Huang Z−2022	11	54	5	48	2.20	[0.70; 6.87]	24.0%	14.9%
Li Z−2022	7	20	2	21	5.12	[0.91; 28.64]	7.2%	10.1%
Qin Y−2018	9	30	1	30	12.43	[1.46; 105.74]	4.0%	7.8%
Shen L−2023	23	32	7	26	6.94	[2.18; 22.12]	12.4%	14.7%
Sun X−2023	6	36	1	32	6.20	[0.70; 54.61]	5.0%	7.6%
Wei F−2020	41	41	12	35	156.04	[8.83; 2756.57]	0.0%	5.1%
Zhao L−2023	5	39	0	33	10.68	[0.57; 200.80]	0.0%	4.9%
Zou Y−2020	10	55	3	58	4.07	[1.06; 15.70]	13.6%	13.0%
Ma C−2022	11	47	5	22	1.04	[0.31; 3.46]	29.8%	14.3%
**Fixed effect model**		**384**		**330**	**5.65**	**[3.53; 9.05]**	**100.0%**	**.**
**Random effects model**					**5.28**	**[2.28; 12.26]**	**.**	**100.0%**

Heterogeneity: $I^2 = 44\%$, $\tau^2 = 0.5932$, $p = 0.07$

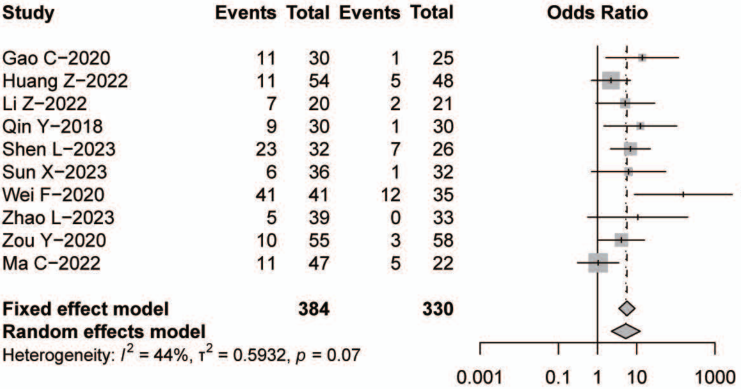

Study	ITCS Events	ITCS Total	FNS Events	FNS Total	OR	95%−CI	Weight (Fixed)	Weight (Random)
Cui X−2023	6	37	3	31	1.81	[0.41; 7.91]	22.3%	18.4%
Xu K−2020	35	55	20	45	2.19	[0.98; 4.89]	65.2%	62.2%
Ye J−2017	2	32	0	32	5.33	[0.25; 115.50]	0.0%	4.3%
Yin Q−2016	8	35	2	35	4.89	[0.96; 24.97]	12.6%	15.1%
**Fixed effect model**		**159**		**143**	**2.60**	**[1.38; 4.92]**	**100.0%**	**.**
**Random effects model**					**2.48**	**[1.30; 4.72]**	**.**	**100.0%**

Heterogeneity: $I^2 = 0\%$, $\tau^2 = 0$, $p = 0.76$

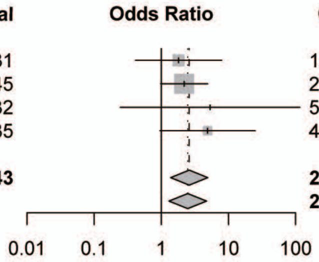

Figure S2 Forest plot of standard pair-wisemeta-analysis for complications.

Study	ITCS Total	ITCS Mean	ITCS SD	FNS Total	FNS Mean	FNS SD	MD	95%-CI	Weight (Fixed)	Weight (rRandom)
Wong Y−2022	26	62.46	8.28	26	48.63	7.15	13.83	[9.62; 18.04]	7.5%	6.6%
Yang Y−2021	19	41.60	12.10	15	39.40	6.30	2.20	[−4.11; 8.51]	3.3%	6.4%
Zhang F−2023	27	105.33	54.97	25	113.00	36.74	−7.67	[−32.92; 17.58]	0.2%	3.1%
Zhou X−2021	30	40.90	5.22	30	42.83	4.69	−1.93	[−4.44; 0.58]	21.1%	6.8%
Ge S−2022	35	37.30	9.90	44	39.20	4.10	−1.90	[−5.40; 1.60]	10.9%	6.7%
Guy R−2023	58	64.19	29.52	56	55.11	30.22	9.08	[−1.89; 20.05]	1.1%	5.6%
He C−2021	36	56.11	12.48	33	49.94	14.46	6.17	[−0.23; 12.57]	3.2%	6.4%
Hu H−2021	24	64.58	18.56	20	79.75	26.35	−15.17	[−28.90; −1.44]	0.7%	5.1%
Lin H−2023	31	43.80	4.10	27	51.70	4.30	−7.90	[−10.07; −5.73]	28.2%	6.8%
Su M−2023	51	49.31	9.44	43	57.33	9.66	−8.02	[−11.90; −4.14]	8.8%	6.7%
Tang Y−2021	45	42.00	11.90	47	52.40	11.00	−10.40	[−15.09; −5.71]	6.0%	6.6%
Tao J−2023	49	130.10	47.90	47	121.00	40.80	9.10	[−8.67; 26.87]	0.4%	4.3%
Wang L−2021	10	58.60	15.50	14	47.40	5.30	11.20	[1.20; 21.20]	1.3%	5.8%
Yan S−2023	27	63.96	22.88	22	67.41	11.65	−3.45	[−13.36; 6.46]	1.4%	5.8%
Yang C−2021	58	88.38	37.60	24	58.92	6.97	29.46	[19.39; 39.53]	1.3%	5.8%
Zhang Y−2022	36	76.81	13.10	33	60.00	12.44	16.81	[10.78; 22.84]	3.7%	6.4%
Zhu Y−2022	32	64.69	24.19	15	88.67	20.24	−23.98	[−37.21; −10.75]	0.8%	5.2%
**Fixed effect model**							**−1.93**	**[−3.08; −0.78]**	**100.0%**	**.**
**Random effects model**	**594**			**521**			**1.31**	**[−5.34; 7.96]**	**.**	**100.0%**

Heterogeneity: $I^2 = 63\%$, $\tau^2 = 141.1793$, $p < 0.01$

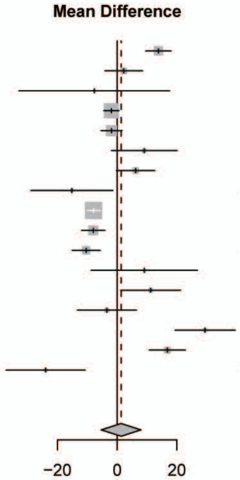

Study	ITCS Total	ITCS Mean	ITCS SD	MBP Total	MBP Mean	MBP SD	MD	95%-CI	Weight (Fixed)	Weight (Random)
Gao C−2020	30	68.73	4.91	25	110.76	5.85	−42.03	[−44.92; −39.14]	28.0%	11.3%
Huang Z−2022	54	36.87	15.39	48	75.35	27.67	−38.48	[−47.32; −29.64]	3.0%	10.9%
Li Z−2022	20	45.16	8.13	21	70.96	9.27	−25.80	[−31.13; −20.47]	8.2%	11.2%
Qin Y−2018	30	73.00	7.83	30	46.50	8.11	26.50	[22.47; 30.53]	14.4%	11.3%
Shen L−2023	32	64.30	17.50	26	100.20	26.00	−35.90	[−47.59; −24.21]	1.7%	10.6%
Sun X−2023	36	88.30	6.48	32	94.76	10.52	−6.46	[−10.67; −2.25]	13.2%	11.3%
Zhao L−2023	39	85.60	10.40	33	112.30	13.50	−26.70	[−32.35; −21.05]	7.3%	11.2%
Zou Y−2020	55	72.56	9.08	58	87.53	9.15	−14.97	[−18.33; −11.61]	20.7%	11.3%
Ma C−2022	47	52.17	11.57	22	92.41	17.87	−40.24	[−48.41; −32.07]	3.5%	11.0%
**Fixed effect model**							**−19.17**	**[−20.70; −17.65]**	**100.0%**	**.**
**Random effects model**	**343**			**295**			**−22.48**	**[−39.46; −5.50]**	**.**	**100.0%**

Heterogeneity: $I^2 = 69\%$, $\tau^2 = 480.6175$, $p < 0.01$

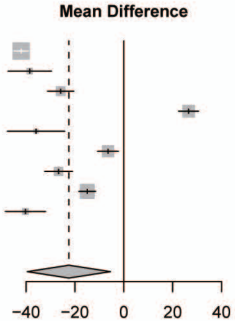

Study	ITCS Total	ITCS Mean	ITCS SD	PCCP Total	PCCP Mean	PCCP SD	MD	95%-CI	Weight (Fixed)	Weight (Random)
Cui X−2023	37	76.27	24.47	31	71.29	20.32	4.98	[−5.67; 15.63]	19.8%	47.7%
Yin Q−2016	35	79.65	11.30	35	96.77	11.26	−17.12	[−22.40; −11.84]	80.2%	52.3%
**Fixed effect model**							**−12.75**	**[−17.48; −8.02]**	**100.0%**	**.**
**Random effects model**	**72**			**66**			**−6.57**	**[−146.83; 133.69]**	**.**	**100.0%**

Heterogeneity: $I^2 = 62\%$, $\tau^2 = 225.8183$, $p < 0.01$

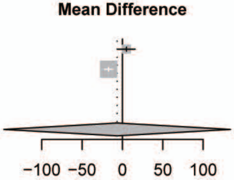

Figure S3 Forest plot of standard pair-wise meta-analysis for operation time.

Study	ITCS Total	ITCS Mean	ITCS SD	FNS Total	FNS Mean	FNS SD	MD	95%−CI	Weight (Fixed)	Weight (Random)
Wong Y−2022	26	21.53	7.49	26	32.96	8.99	−11.43	[−15.93; −6.93]	8.3%	6.0%
Yang J−2021	31	17.30	6.20	28	27.80	6.30	−10.50	[−13.70; −7.30]	16.4%	6.1%
Yang Y−2021	19	34.80	26.00	15	52.40	10.40	−17.60	[−30.42; −4.78]	1.0%	5.6%
Zhang F−2023	27	10.00	7.07	25	49.20	33.66	−39.20	[−52.66; −25.74]	0.9%	5.5%
Zhou X−2021	30	30.27	9.04	30	99.73	52.73	−69.46	[−88.60; −50.32]	0.5%	5.0%
Ge S−2022	35	35.10	23.40	44	46.20	14.90	−11.10	[−20.02; −2.18]	2.1%	5.8%
Guy R−2023	58	35.69	29.15	56	35.71	29.66	−0.02	[−10.82; 10.78]	1.4%	5.7%
He C−2021	36	56.11	12.48	33	49.94	14.46	6.17	[−0.23; 12.57]	4.1%	6.0%
Hu H−2021	24	23.71	28.13	20	69.45	50.47	−45.74	[−70.56; −20.92]	0.3%	4.4%
Lin H−2023	31	41.50	5.70	27	50.30	4.30	−8.80	[−11.38; −6.22]	25.2%	6.1%
Su M−2023	51	46.47	22.88	43	56.05	18.66	−9.58	[−17.98; −1.18]	2.4%	5.9%
Tang Y−2021	45	47.30	9.30	47	50.60	10.60	−3.30	[−7.37; 0.77]	10.1%	6.1%
Tao J−2023	49	77.60	98.30	47	119.60	97.60	−42.00	[−81.19; −2.81]	0.1%	3.1%
Wang L−2021	10	33.30	25.70	14	50.60	10.30	−17.30	[−34.12; −0.48]	0.6%	5.2%
Yan S−2023	27	20.67	4.71	22	50.45	5.28	−29.78	[−32.61; −26.95]	20.9%	6.1%
Yang C−2021	58	88.38	37.60	24	58.92	6.97	29.46	[19.39; 39.53]	1.7%	5.8%
Zhang B−2022	34	55.10	19.10	31	57.20	21.50	−2.10	[−12.02; 7.82]	1.7%	5.8%
Zhu Y−2022	32	18.59	10.79	15	53.33	14.96	−34.74	[−43.18; −26.30]	2.4%	5.9%
**Fixed effect model**							**−13.15**	**[−14.45; −11.86]**	**100.0%**	**.**
**Random effects model**	**623**			**547**			**−16.04**	**[−27.00; −5.07]**	**.**	**100.0%**

Heterogeneity: $I^2 = 67\%$, $\tau^2 = 422.0884$, $p < 0.01$

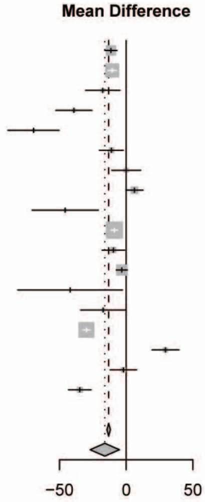

Study	ITCS Total	ITCS Mean	ITCS SD	MBP Total	MBP Mean	MBP SD	MD	95%−CI	Weight (Fixed)	Weight (Random)
Gao C−2020	30	69.47	5.15	25	110.92	10.10	−41.45	[−45.82; −37.08]	27.7%	11.2%
Huang Z−2022	54	21.86	18.19	48	153.45	64.27	−131.59	[−150.41; −112.77]	1.5%	11.0%
Li Z−2022	20	61.63	13.82	21	138.52	21.04	−76.89	[−87.74; −66.04]	4.5%	11.1%
Qin Y−2018	30	59.80	10.10	30	150.50	28.20	−90.70	[−101.42; −79.98]	4.6%	11.1%
Shen L−2023	32	30.00	9.20	26	153.10	22.60	−123.10	[−132.35; −113.85]	6.2%	11.2%
Sun X−2023	36	126.45	12.34	32	155.88	15.64	−29.43	[−36.18; −22.68]	11.6%	11.2%
Zhao L−2023	39	85.60	10.40	33	112.30	13.50	−26.70	[−32.35; −21.05]	16.6%	11.2%
Zou Y−2020	55	118.98	10.55	58	149.11	13.64	−30.13	[−34.61; −25.65]	26.3%	11.2%
Ma C−2022	47	51.81	19.21	22	265.23	53.15	−213.42	[−236.30; −190.54]	1.0%	10.8%
**Fixed effect model**							**−46.62**	**[−48.92; −44.32]**	**100.0%**	**.**
**Random effects model**	**343**			**295**			**−84.23**	**[−132.27; −36.18]**	**.**	**100.0%**

Heterogeneity: $I^2 = 65\%$, $\tau^2 = 3826.2274$, $p < 0.01$

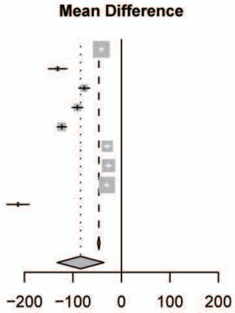

Study	ITCS Total	ITCS Mean	ITCS SD	PCCP Total	PCCP Mean	PCCP SD	MD	95%−CI	Weight (Fixed)	Weight (Random)
Cui X−2023	37	111.19	52.44	31	121.71	53.82	−10.52	[−35.91; 14.87]	4.2%	4.2%
Yin Q−2016	35	79.65	11.30	35	96.77	11.26	−17.12	[−22.40; −11.84]	95.8%	95.8%
**Fixed effect model**							**−16.85**	**[−22.02; −11.67]**	**100.0%**	**.**
**Random effects model**	**72**			**66**			**−16.85**	**[−22.02; −11.67]**	**.**	**100.0%**

Heterogeneity: $I^2 = 0\%$, $\tau^2 = 0$, $p = 0.62$

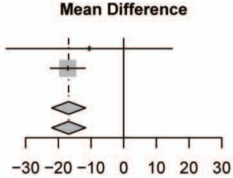

Figure S4 Forest plot of standard pair-wise meta-analysis for intraoperative bleeding volume.

Study	ITCS Events	ITCS Total	FNS Events	FNS Total	OR	95%-CI	Weight (Fixed)	Weight (Random)
Wong Y−2022	5	26	3	26	1.83	[0.39; 8.59]	9.7%	9.7%
Yang J−2021	2	31	0	28	4.83	[0.22; 105.07]	2.5%	2.5%
Zhang F−2023	9	27	4	25	2.62	[0.69; 9.98]	13.1%	13.1%
Zhou X−2021	1	30	0	30	3.10	[0.12; 79.23]	2.2%	2.2%
Ge S−2022	3	35	4	44	0.94	[0.20; 4.49]	9.5%	9.5%
Guy R−2023	9	58	3	56	3.24	[0.83; 12.68]	12.6%	12.6%
He C−2021	2	36	0	33	4.86	[0.22; 104.94]	2.5%	2.5%
Hu H−2021	6	24	3	20	1.89	[0.41; 8.78]	9.9%	9.9%
Lin H−2023	3	31	1	27	2.79	[0.27; 28.50]	4.3%	4.3%
Su M−2023	10	51	3	43	3.25	[0.83; 12.69]	12.6%	12.6%
Tang Y−2021	3	45	2	47	1.61	[0.26; 10.10]	6.9%	6.9%
Tao J−2023	4	49	1	47	4.09	[0.44; 38.01]	4.7%	4.7%
Yan S−2023	2	27	0	22	4.41	[0.20; 96.84]	2.4%	2.4%
Yang C−2021	3	58	0	24	3.09	[0.15; 62.13]	2.6%	2.6%
Zhang B−2022	1	34	0	31	2.82	[0.11; 71.84]	2.2%	2.2%
Zhang Y−2022	1	36	0	33	2.83	[0.11; 71.94]	2.2%	2.2%
**Fixed effect model**		**598**		**536**	**2.45**	**[1.51; 3.97]**	**100.0%**	**.**
**Random effects model**					**2.45**	**[1.94; 3.10]**	**.**	**100.0%**

Heterogeneity: $I^2 = 0\%$, $\tau^2 = 0$, $p = 1.00$

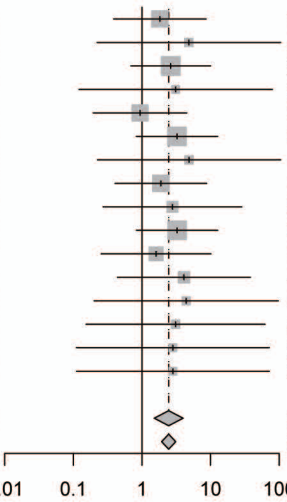

Study	ITCS Events	ITCS Total	MBP Events	MBP Total	OR	95%-CI	Weight (Fixed)	Weight (Random)
Gao C−2020	5	30	1	25	4.80	[0.52; 44.15]	5.5%	6.6%
Huang Z−2022	11	54	5	48	2.20	[0.70; 6.87]	25.4%	22.1%
Li Z−2022	3	20	2	21	1.68	[0.25; 11.27]	10.0%	8.8%
Qin Y−2018	4	30	1	30	4.46	[0.47; 42.51]	5.2%	6.4%
Shen L−2023	9	32	5	26	1.64	[0.47; 5.70]	23.9%	19.0%
Sun X−2023	6	36	1	32	6.20	[0.70; 54.61]	5.3%	6.8%
Wei F−2020	24	41	0	35	99.40	[5.70; 1731.99]	0.0%	4.0%
Zhao L−2023	5	39	0	33	10.68	[0.57; 200.80]	0.0%	3.8%
Zou Y−2020	6	55	2	58	3.43	[0.66; 17.77]	10.4%	11.5%
Ma C−2022	6	47	2	22	1.46	[0.27; 7.91]	14.3%	11.0%
**Fixed effect model**		**384**		**330**	**4.12**	**[2.45; 6.91]**	**100.0%**	**.**
**Random effects model**					**3.00**	**[1.53; 5.87]**	**.**	**100.0%**

Heterogeneity: $I^2 = 5\%$, $\tau^2 = 0.0632$, $p = 0.40$

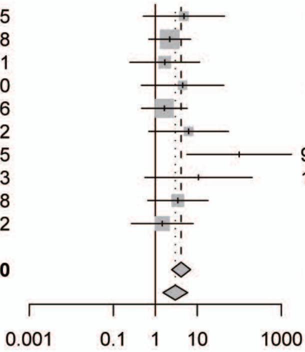

Study	ITCS Events	ITCS Total	MBP Events	MBP Total	OR	95%-CI	Weight (Fixed)	Weight (Random)
Cui X−2023	3	37	3	31	0.82	[0.15; 4.40]	36.4%	26.0%
Xu K−2020	11	55	4	45	2.56	[0.76; 8.69]	42.7%	49.1%
Yin Q−2016	5	35	2	35	2.75	[0.50; 15.25]	20.8%	24.9%
**Fixed effect model**		**127**		**111**	**1.97**	**[0.85; 4.54]**	**100.0%**	**.**
**Random effects model**					**1.94**	**[0.41; 9.14]**	**.**	**100.0%**

Heterogeneity: $I^2 = 0\%$, $\tau^2 = 0$, $p = 0.51$

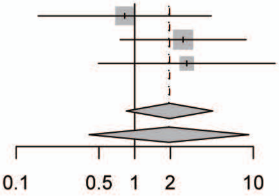

Figure S5 Forest plot of standard pair-wise meta-analysis for femoral head necrosis and nonunion.

Study	ITCS Total	ITCS Mean	ITCS SD	FNS Total	FNS Mean	FNS SD	MD	95%-CI	Weight (Fixed)	Weight (Random)
Wong Y−2022	26	5.12	2.43	26	4.22	1.81	0.90	[−0.26; 2.06]	0.3%	3.2%
Yang J−2021	31	4.40	0.90	28	3.60	0.90	0.80	[0.34; 1.26]	1.6%	5.6%
Yang Y−2021	19	3.80	0.77	15	3.83	0.65	−0.03	[−0.51; 0.45]	1.5%	5.6%
Zhang F−2023	27	5.07	1.54	25	3.72	0.98	1.35	[0.65; 2.05]	0.7%	4.7%
Zhou X−2021	30	6.03	1.45	30	5.23	1.33	0.80	[0.10; 1.50]	0.7%	4.7%
Ge S−2022	35	3.52	0.68	44	3.45	0.40	0.07	[−0.18; 0.32]	5.3%	6.2%
Guy R−2023	58	5.10	0.81	56	2.86	0.77	2.24	[1.95; 2.53]	4.0%	6.1%
He C−2021	36	3.27	0.42	33	3.22	0.37	0.05	[−0.14; 0.24]	9.8%	6.4%
Hu H−2021	24	4.14	1.01	20	3.53	0.90	0.61	[0.05; 1.17]	1.1%	5.2%
Lin H−2023	31	5.97	0.16	27	5.91	0.19	0.06	[−0.03; 0.15]	41.0%	6.5%
Su M−2023	51	4.71	0.94	43	3.88	0.70	0.83	[0.50; 1.16]	3.1%	6.0%
Tang Y−2021	45	4.00	0.39	47	2.97	0.35	1.03	[0.88; 1.18]	14.8%	6.4%
Wang L−2021	10	3.90	1.84	14	3.85	0.51	0.05	[−1.12; 1.22]	0.2%	3.2%
Yan S−2023	27	4.46	0.59	22	3.76	0.42	0.70	[0.42; 0.98]	4.2%	6.2%
Yang C−2021	58	2.97	1.30	24	1.67	0.34	1.30	[0.94; 1.66]	2.6%	5.9%
Zhang B−2022	34	2.36	0.68	31	2.26	0.82	0.10	[−0.27; 0.47]	2.5%	5.9%
Zheng H−2022	18	2.82	0.51	18	2.47	0.44	0.35	[0.04; 0.66]	3.5%	6.1%
Zhu Y−2022	32	3.31	0.69	15	2.73	0.46	0.58	[0.25; 0.91]	3.1%	6.0%
**Fixed effect model**							**0.43**	**[0.38; 0.49]**	**100.0%**	**.**
**Random effects model**	**592**			**518**			**0.65**	**[0.35; 0.96]**	**.**	**100.0%**

Heterogeneity: $I^2 = 69\%$, $\tau^2 = 0.3317$, $p < 0.01$

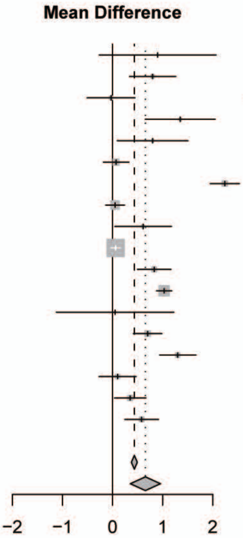

Study	ITCS Total	ITCS Mean	ITCS SD	MBP Total	MBP Mean	MBP SD	MD	95%-CI	Weight (Fixed)	Weight (Random)
Gao C−2020	30	5.03	0.74	25	4.37	0.56	0.66	[0.32; 1.00]	4.6%	15.4%
Huang Z−2022	54	3.65	1.85	48	3.65	1.85	0.00	[−0.72; 0.72]	1.0%	7.8%
Li Z−2022	20	5.11	1.25	21	4.72	1.14	0.39	[−0.34; 1.12]	1.0%	7.6%
Qin Y−2018	30	5.48	1.31	30	4.33	0.88	1.15	[0.59; 1.71]	1.7%	10.3%
Shen L−2023	32	5.51	2.59	26	5.04	1.42	0.47	[−0.58; 1.52]	0.5%	4.5%
Sun X−2023	36	4.34	1.15	32	3.38	0.51	0.96	[0.54; 1.38]	3.1%	13.6%
Zhao L−2023	39	4.26	0.23	33	3.40	0.14	0.86	[0.77; 0.95]	72.4%	21.3%
Zou Y−2020	55	5.72	0.53	58	4.49	0.48	1.23	[1.04; 1.42]	15.6%	19.4%
**Fixed effect model**							**0.90**	**[0.83; 0.97]**	**100.0%**	**.**
**Random effects model**	**296**			**273**			**0.82**	**[0.51; 1.14]**	**.**	**100.0%**

Heterogeneity: $I^2 = 71\%$, $\tau^2 = 0.0748$, $p < 0.01$

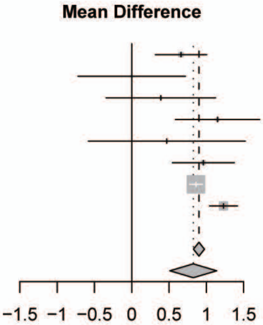

Study	ITCS Total	ITCS Mean	ITCS SD	PCCP Total	PCCP Mean	PCCP SD	MD	95%-CI	Weight (Fixed)	Weight (Random)
Cui X−2023	37	7.10	0.88	31	6.10	0.91	1.00	[0.57; 1.43]	21.9%	24.8%
Xu K−2020	55	5.04	1.00	45	4.64	0.74	0.40	[0.06; 0.74]	34.3%	28.2%
Ye J−2017	32	3.39	0.77	32	3.20	0.66	0.19	[−0.16; 0.54]	32.1%	27.7%
Yin Q−2016	35	6.96	1.51	35	6.17	0.92	0.79	[0.20; 1.38]	11.7%	19.2%
**Fixed effect model**							**0.51**	**[0.31; 0.71]**	**100.0%**	**.**
**Random effects model**	**159**			**143**			**0.57**	**[−0.03; 1.16]**	**.**	**100.0%**

Heterogeneity: $I^2 = 68\%$, $\tau^2 = 0.0961$, $p = 0.02$

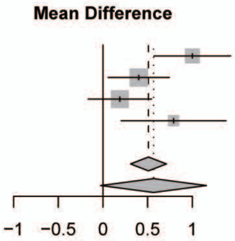

Figure S6 Forest plot of standard pair-wise meta-analysis for fracture healing time.

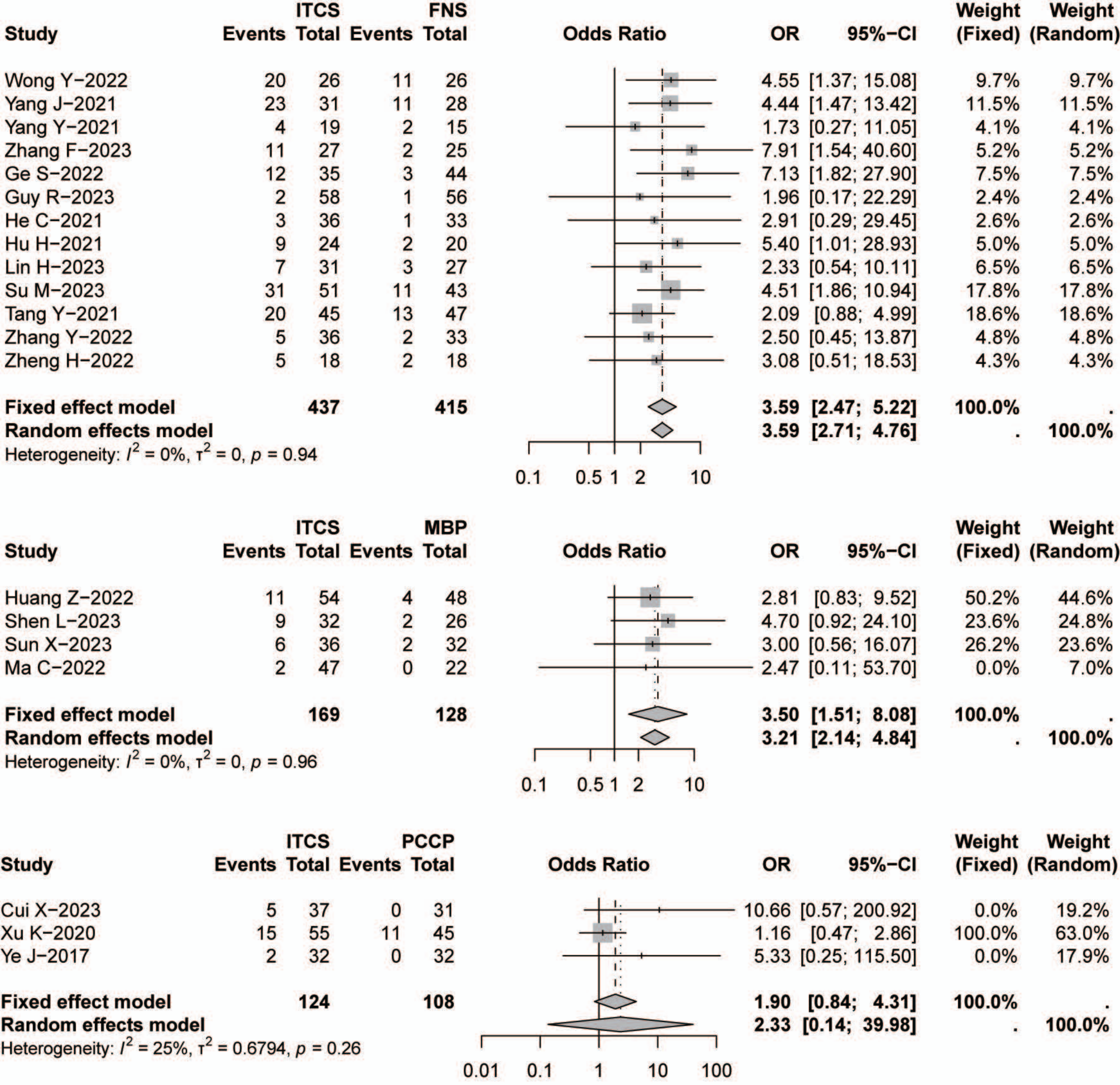

Figure S7 Forest plot of standard pair-wise meta-analysis for femoral neck shortening.
